# Effect of Al Incorporation on the Structural and Optical Properties of Sol–Gel AZO Thin Films

**DOI:** 10.3390/ma16093329

**Published:** 2023-04-24

**Authors:** Hermine Stroescu, Madalina Nicolescu, Daiana Mitrea, Ecaterina Tenea, Irina Atkinson, Mihai Anastasescu, Jose Maria Calderon-Moreno, Mariuca Gartner

**Affiliations:** “Ilie Murgulescu” Institute of Physical Chemistry, Romanian Academy, 202 Spl. Independentei, 060021 Bucharest, Romania

**Keywords:** Al-doped ZnO, sol–gel, optical properties, Raman spectroscopy, photoluminescence

## Abstract

ZnO and Al-doped ZnO (AZO) thin films were prepared using the sol–gel method and deposited on a Silicon (Si(100)) substrate using the dipping technique. The structure, morphology, thickness, optical constants in the spectral range 300–1700 nm, bandgap (E_g_) and photoluminescence (PL) properties of the films were analyzed using X-ray diffractometry (XRD), X-ray fluorescence (XRF), atomic force microscopy (AFM), scanning electron microscopy (SEM), spectroscopic ellipsometry (SE), Raman analysis and PL spectroscopy. The results of the structure and morphology analyses showed that the thin films are polycrystalline with a hexagonal wurtzite structure, as well as continuous and homogeneous. The PL background and broader peaks observable in the Raman spectra of the AZO film and the slight increase in the optical band gap of the AZO thin film, compared to undoped ZnO, highlight the effect of defects introduced into the ZnO lattice and an increase in the charge carrier density in the AZO film. The PL emission spectra of the AZO thin film showed a strong UV line corresponding to near-band-edge ZnO emission along with weak green and red emission bands due to deep-level defects, attributed to the oxygen-occupied zinc vacancies (O_Zn_ lattice defects).

## 1. Introduction

Over time, ZnO has received tremendous attention as a result of its interesting properties, such as a wide band gap of 3.37 eV, remarkable electron mobility (1 to 200 cm^2^/Vs) and large exciton binding energy of 60 meV, and it is considered to be a promising candidate for a wide range of applications [1]. Due to its versatility, high thermal stability, biocompatibility and also accessibility and low cost of preparation, ZnO was explored in different forms (bulk, films, nanomaterials, etc.) [2]. In particular, ZnO thin films are used for the development of optoelectronic [3], transparent conductive oxides (TCOs) [4] and piezoelectric devices [5], photovoltaics [6], photocatalysts [7], gas sensors [8,9,10] or biosensors [11,12,13] due to their impressive optical (photoluminescence—PL) [14,15,16], electrical (thermoelectric, piezoelectric) [17,18,19,20] and biological (antimicrobial and antibacterial) [21,22,23] properties. It is well known that by doping, the aforementioned properties of ZnO thin films can be significantly improved. For this purpose, the elements of II, III, IV groups are widely used, as they increase the electrical and optical performance of ZnO-based materials [24,25,26,27]. Among them, the group III elements such as B, Al, Ga and In are frequently used as extrinsic donors to achieve a higher conductivity, transparency and a better quality of the resulting doped films by promoting a free electron through the substitution of zinc cation with a trivalent ion [28,29]. Many studies feature the Al-doped ZnO thin films (AZO) as an efficient alternative to ITO films due to their facile preparation and the low cost of the raw materials [30,31,32]. AZO films can be manufactured by different deposition methods such as sol–gel [30,33,34,35], spray pyrolysis [27], chemical bath deposition [36], RF sputtering [37,38], atomic layer [39] and pulsed laser deposition [40]. The sol–gel synthesis displays a series of advantages such as cost effectiveness, ease of preparation, stoichiometry, homogeneity, viscosity and thickness control, lower working temperatures and the use of a higher concentration of dopant [41]. AZO-based materials have been investigated in different fields, having applicability as a transparent conductive layer for flexible (polymer dispersed-liquid crystals) or rigid (liquid crystals) display devices [42], optoelectronics [35], thin film transistors [43], solar cells [44,45], oxygen [46], glucose [47], humidity [48] and UV sensors [49,50]. Additionally, the AZO films were also investigated toward the PL properties at room temperature [29,31,51,52,53,54,55,56]. The PL spectra display characteristic features corresponding to the UV and visible emissions. The UV band can be assigned to the near-band-edge (NBE) emission or to the related recombination of free excitons, while the visible bands are ascribed to the defects in ZnO, such as oxygen vacancies (V_O_), zinc vacancies (V_Zn_), Zn interstitials (Zn_i_) and oxygen atoms at zinc position in crystal lattices (O_Zn_) [57]. According to the literature, the performance of AZO thin films was explored by varying several parameters such as substrate [58,59,60] and solvent type [29,52], dopant concentration [34,61], annealing temperature [62] and thickness (number of layers) [31]. It was observed that the PL properties are strongly influenced by the previously mentioned factors, especially by the type of substrate used [63], amount of dopant [54,55,56] and annealing temperature [64]. A more recent paper reported by Patel et al. [60] represents a review of AZO thin films prepared by different methods and deposited on different substrates to demonstrate their influence on the film properties. It was shown that choosing the suitable method and substrate types leads to specific properties. The structural, optical and electrical properties of aluminum-doped ZnO thin films mainly prepared by the sputtering technique were found to be affected by several parameters (power, time, temperature, working pressure, gas mixture or dopant concentration) and also by the substrate used (glass or silicon). The use of AZO films as possible candidates for the development of UV sensors through a spin-coating sol–gel preparation was reported by Lee et al. [55]. An improvement of the responsivity of UV sensors was observed for the aluminum-doped films compared to the undoped sample.

This paper presents a comprehensive evaluation of the structural, morphological, optical and PL properties of the AZO thin film deposited by the sol–gel dip-coating method on Silicon (Si(100)) substrate. Our aim was to evaluate the influence of the aluminum incorporation of the aforementioned properties in comparison with the undoped ZnO and taking into account the related literature studies.

## 2. Materials and Methods

### 2.1. Film Preparation

**Figure 1:**
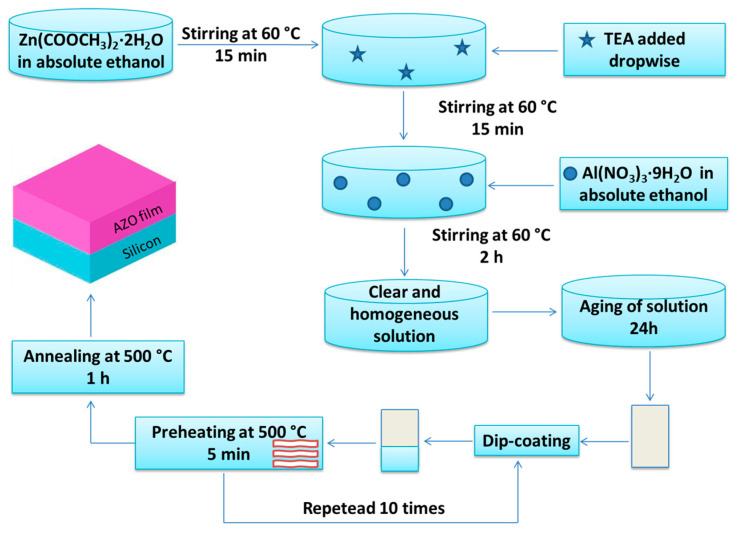
The flow chart of the thin films’ preparation.

The sol–gel dip-coating method was chosen for the preparation of ZnO and AZO films deposited on Si(100) substrate (Figure 1) by using reagents including zinc acetate di-hydrate-Zn (CH_3_COO)_2_·2H_2_O (ZAD), aluminum nitrate nonahydrate Al(NO_3_)_3_·9H_2_O (ANN), absolute ethyl alcohol-CH_3_CH_2_OH and triethanolamine-N(CH_2_-CH_2_OH)(TEA). All chemicals were purchased from Merck (New York, NY, USA).

By dissolving the appropriate amount of ZAD and ANN into absolute ethanol, the corresponding zinc acetate and aluminum nitrate solutions of 0.1 M were obtained. For the preparation of the undoped ZnO thin films, the zinc acetate solution was kept at 60 °C under continuous stirring for 15 min, followed by the dropwise addition of TEA, in a molar ratio of TEA/ZAD = 1/5. Following the same procedure described above, the AZO thin films were further synthetized by adding the suitable quantity of the as-prepared aluminum nitrate solution to achieve the Al-Zn sol, in which the aluminum content was 0.5 at. %. The clear and homogeneous solutions obtained after 2 h of stirring at 60 °C were aged at room temperature for 1 day before using them in the dip-coating process. Pure ZnO and AZO final films were obtained by repeating the deposition step ten times. The films were preheated at 500 °C for 5 min, with a heating rate of 5 °C/min after each deposition. The final annealing process of the undoped and doped ZnO films was performed at 500 °C for 1 h.

### 2.2. Characterization Methods

XRF—Elemental analysis of the film was performed in vacuum using a Rigaku ZSX Primus II spectrometer (Tokyo, Japan). The results were analyzed using EZ-scan combined with Rigaku SQX fundamental parameters software (standard less) which is capable of automatically correcting all matrix effects, including line overlaps.

XRD analysis was used to investigate the crystallinity of the film deposited on the Si substrate. XRD pattern was collected using a Rigaku Ultima IV diffractometer with Cu K radiation (λ = 1.5406), operating at 40 kV and 30 mA, and equipped with a thin film attachment for grazing incidence X-ray measurement at an incidence angle of 0.5°. XRD pattern was recorded with a scan step of 0.02° and a scan speed of 5°/min. The phase identification and lattice parameter calculation were performed using Rigaku’s PDXL software connected to the ICDD database.

The average crystallite size of ZnO and AZO films using (100), (002) and (101) crystal planes were estimated according to Scherrer’s Equation (1) along (002) direction:

(1) D=kλ/(βcosθ)

where k is Scherrer’s constant, usually taken as 0.90, λ is the X-ray wavelength, β is the full width at half maximum of the diffraction line (FWHM), and θ is the diffraction angle.

AFM—Morphology and roughness of the AZO films were evaluated using atomic force microscopy (AFM). The measurements were made in true non-contact™ mode with XE-100 (Park Systems). All AFM images were recorded with sharp tips, NCHR (Nanosensors™), of <8 nm tip apex, app. 125 µm length, 30 µm width, spring constant ~42 N/m and ~330 kHz resonance frequency. The AFM images were processed with XEI program (v 1.8.0—Park Systems) for 1st order tilt correction and are presented in so-called “enhanced contrast” view mode.

SEM—The morphology of the films was investigated by scanning electron microscopy (SEM) in a Quanta 3D FEG apparatus with an Everhart–Thornley secondary electron detector, operating between 5 and 30 kV.

SE—The AZO thin films deposited on Si were analyzed by spectroscopic ellipsometry (SE) in 300–1700 nm range, at an incidence angle of 70°, performed on VASE-Woollam equipment from J. A. Woollam Co. Inc. (Lincoln, NE, USA). The analysis of the experimental ellipsometric parameters (ψ and Δ) was performed with the commercially available WVASE32 software package (v. 3.920, J. A. Woollam Co. Inc.).

Raman and PL spectra were recorded in a Horiba Jobin Yvon LabRam HR spectrometer using a 325 nm excitation laser and a NUV 40× objective.

## 3. Results and Discussion

### 3.1. X-ray Fluorescence (XRF)

**Figure 2:**
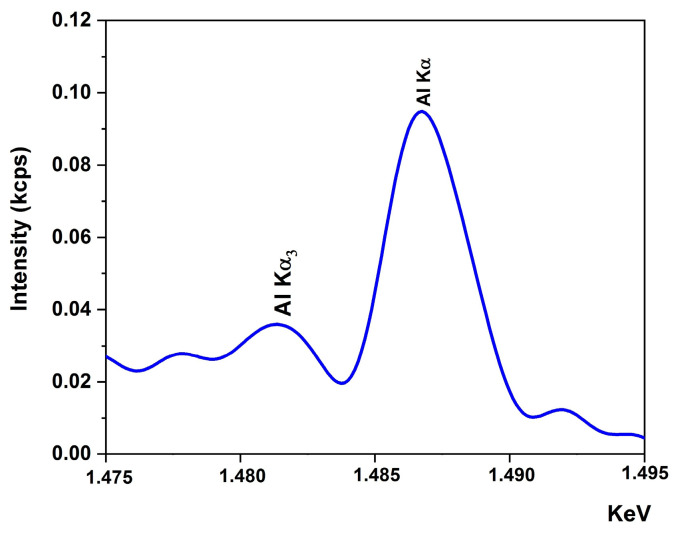
X-ray fluorescence spectrum of Al element.

The dopant concentration and elemental composition of AZO film were determined using XRF analysis. Figure 2 illustrates the XRF spectrum of Al. The presence of the Al Kα (1.487 KeV) and Al Kα3 (1.482 KeV) spectral lines highlighted the presence of Al. Table 1 shows that the Al content of AZO film of 0.65 at. % is slightly higher than the targeted concentration for AZO film (0.5 at. %). Similarly, Singh and Mukherjee [65] observed a small increase in Al content with the AZO film thickness (2.93 at. % against the theoretical value of 2 at. % for a 5.1 μm thickness).

### 3.2. Structure and Morphology

#### 3.2.1. XRD

**Figure 3:**
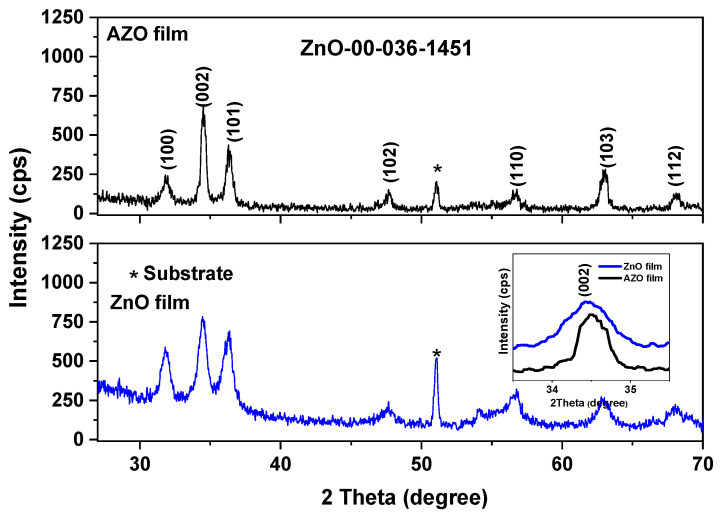
XRD diffractograms of pure ZnO and AZO films deposited on Si (the inset graph represents the magnification of the (002) crystal plane); *—diffraction line corresponding to the substrate.

Figure 3 illustrates the XRD patterns of pure ZnO and AZO thin films deposited on the Si substrate. According to the JCPDS Card no 00-036-1451, the diffraction lines in both XRD patterns originated from (100), (002), (101), (102), (110), (103) and (112) reflections of hexagonal ZnO crystal structure. The sharp diffraction line at about 51° can be assigned to the Si substrate. No diffraction lines corresponding to metallic Al or other Al compounds were detected, suggesting the incorporation of Al into the ZnO lattice.

A slight orientation along the *c*-axis (002) was observed for AZO film (Figure 3), associated with reduced internal stress and surface energy [66]. The inset graph in Figure 3 illustrates the (002) crystal plane of both films in the 2Θ region from 33.5° to 35.5°. The observed shift toward the higher angle value of the diffraction lines of the AZO film compared to that of pure ZnO film can be related to the decrease in lattice parameters. The decreasing in the lattice parameters for AZO film can also be explained by the fact that Al^3+^ with a smaller ionic radius (rAl^3+^ = 0.54 Å) substituted Zn^2+^ (rZn^2+^ = 0.74 Å) that reduces ZnO lattice (Table 2).

The average crystallite size of both films was also estimated using the XRD data, and the results are listed in Table 2. The average crystallite size of AZO film was found to be 212 Å compared to 115 Å for pure ZnO film. Blagoev et al. [67] reported a similar tendency of the crystallite size for the AZO film obtained using the atomic layer deposition with low Al content.

#### 3.2.2. AFM

**Figure 4:**
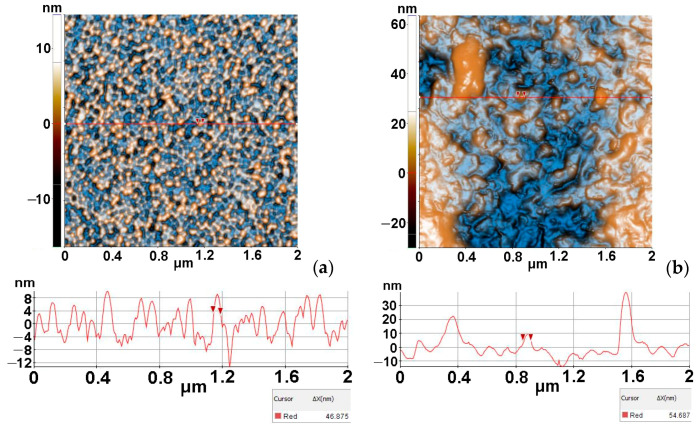
Two-dimensional-enhanced contrast AFM images, scanned over an area of (2 µm × 2 µm) together with representative surface profiles (below each image), for undoped ZnO (**a**) and, respectively, AZO films deposited on Si (**b**).

Figure 4 presents the morphology of the undoped ZnO (Figure 4a) and of the AZO films deposited on Si (Figure 4b), based on 2D-enhanced contrast AFM images scanned over an area of (2 µm × 2 µm).

The morphology of the ZnO film deposited on Si (Figure 4a) is uniform, consisting of quasi-spherical small particles with diameters of tens of nm, with the selected particle along the corresponding line being ~47 nm. The film is smooth as its Z-scale exhibits a vertical corrugation of ~20 nm (from −12 to +8 nm).

In contrast, the AZO film deposited on Si (Figure 4b) exhibits a more corrugated surface, with the agglomeration of particles (rippled aspect). The corresponding line-scan has a vertical height profile of ~40 nm (from −10 to +30 nm), with non-uniform hills and valleys. One of the smallest surface particles, marked along the line-scan in Figure 4b, has a diameter of ~55 nm. The undoped ZnO film deposited on Si exhibits a uniform distribution of the particles (bright yellow spots—Figure 4a) and superficial pores (blue dark spots) in comparison with the Al-doped film, which is less uniform, more corrugated and more compact.

**Figure 5:**
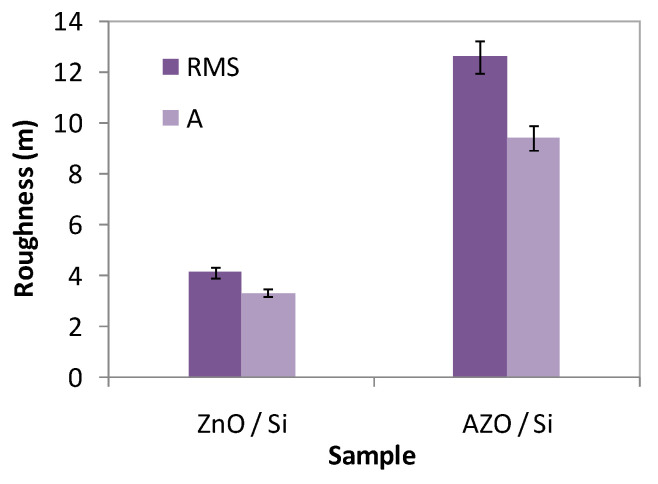
Comparative root mean square (RMS) and average (A) roughness histograms for the AZO film in comparison with undoped ZnO.

The ZnO/Si film has a global RMS roughness, on the whole scanned scale, of 4.14 nm, while the AZO/Si film presents a RMS roughness of 12.6 nm (Figure 5).

#### 3.2.3. SEM

**Figure 6:**
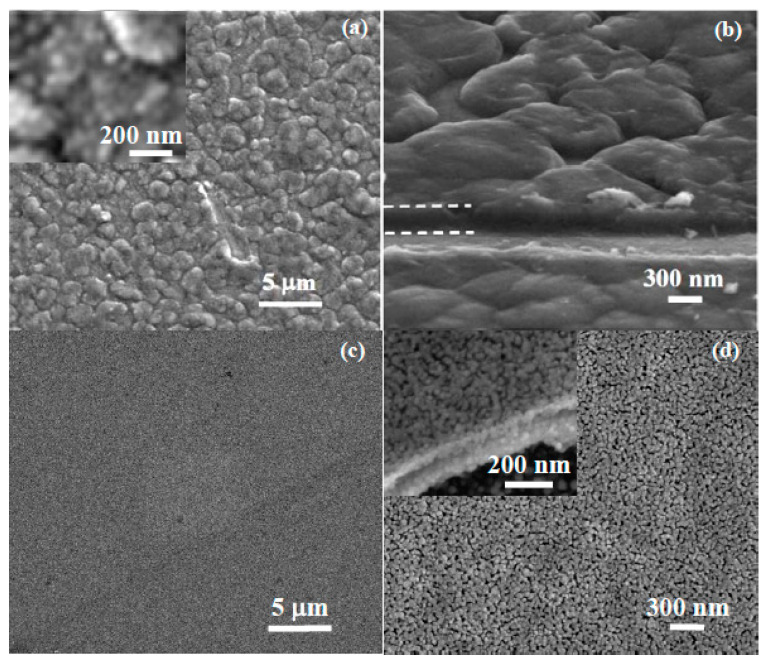
SEM images showing the surface morphology and thickness of AZO films (**a**,**b**) and ZnO (**c**,**d**), recorded at different magnifications.

Figure 6 shows SEM micrographs of the AZO and ZnO films deposited on Si. The AZO film shows microscale irregular aggregates, typically 1–2 microns in size (Figure 6a). These aggregates consist of nanoparticles with sizes below 50 nm, revealed in the top view images of the film surface at a higher magnification (inset in Figure 6a). The film thickness was estimated to be approximately 300 nm (in agreement with that calculated from SE) from edge view images of scratched areas of the film, as shown in Figure 6b, where dashed lines have been added as guide to the eye at the edge of the film. The ZnO film (Figure 6c,d) consists of nanoscale grains also with nanosized intergranular porosity; therefore, the morphology of the ZnO film shows finer and more homogeneous features than the AZO film. The ZnO film is thinner, about 200 nm (inset in Figure 6d), confirming the SE thickness results.

### 3.3. Optical Properties

#### 3.3.1. SE

SE measurements were performed to obtain information about the optical properties of the sol–gel undoped ZnO and AZO thin films.

The design used in the regression analysis was a three-layer model consisting of surface roughness layer/ZnO or AZO film/substrate.

The thickness of the roughness layer was modeled by the Bruggeman effective medium approximation (BEMA), assuming a mixture of 50% film and 50% voids. The Tauc–Lorentz model was used to describe the optical properties of the ZnO and AZO films for the energies around the bandgap.

**Figure 7:**
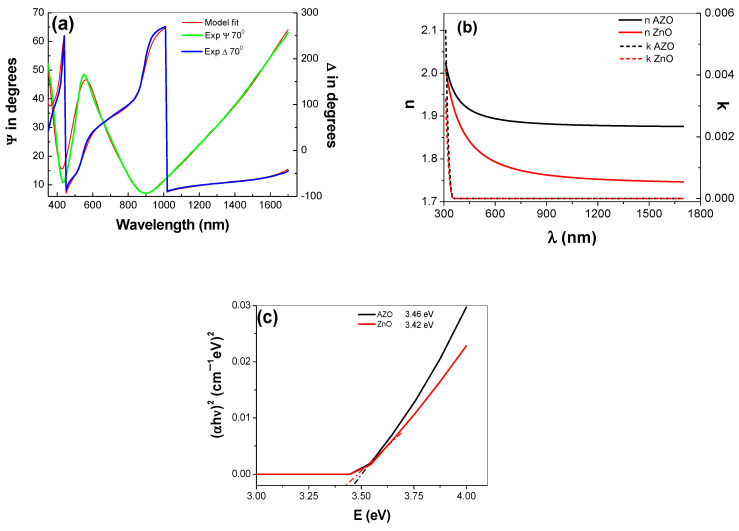
Experimental and calculated spectra of ellipsometric parameters (ψ, Δ), for AZO films deposited on Si (**a**), the optical constants (n, k) for AZO vs. ZnO films deposited on Si (**b**) and the optical bandgap energy (E_g_) for AZO vs. ZnO (**c**).

The goodness of the data fitting with the chosen model is evidenced by the low value of the mean square error (MSE) [68,69] and the overlap between the experimental and calculated points (Figure 7a) for ellipsometric parameters Ψ and Δ. The optical constants (n, k) (Figure 7b), the film thickness (d_film_), the roughness thickness (d_rough_) and the optical band gap (E_g_) were obtained from the best fit, presented further in Table 3.

The value of E_g_ was calculated using the Tauc equation [70]:

(2) $$\alpha h\nu =A{\left(h\nu +E_{g}\right)}^{n}$$

where α is the absorption coefficient and its values are derived from the extinction coefficient, k, (α=4πk/λ), hν is the photon energy, A is a constant and n is ½ for allowed direct band gap.

The energy gap (E_g_) value was calculated by extrapolation with a straight line in the plot (αhν)12 vs. photon energy (hν) (Figure 7c).

The ellipsometric parameters values increase after doping, e.g., the refractive index increased from 1.78 to 1.89 and the band gap changed from 3.42 to 3.45, respectively.

The widened optical band gap (E_g_) of AZO thin film obtained (in our work) is in good agreement with Pelicano et al. [71]. They reported that the enlargement of the optical band gap is due to an increase in charge carrier density in the AZO samples [71].

#### 3.3.2. Raman

**Figure 8:**
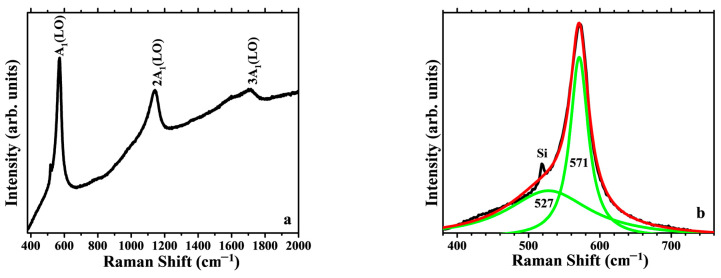

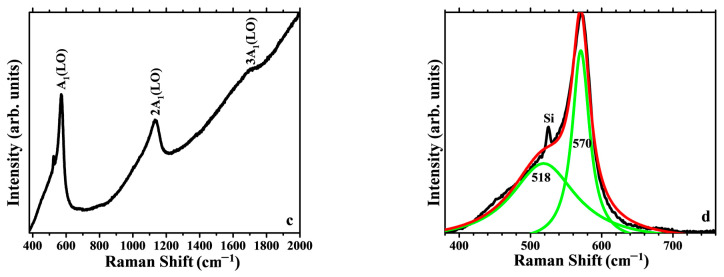
Raman spectra of ZnO and AZO thin films deposited on Si, in the spectral region 400–2000 cm^−1^ (**a**,**c**), and deconvolution (green curves) of the main band in the 400–700 cm^−1^ region for ZnO (**b**) and AZO (**d**).

Figure 8 shows the resonant Raman spectra of ZnO and AZO films deposited on Si, collected using UV excitation (325 nm, 3.81 eV), dominated by the presence of the A_1_(LO) mode of ZnO at ~570 cm^−1^ and its overtones at multiple wavenumbers. The second order 2A_1_(LO) appears at ~1140 cm^−1^ (Figure 8a,c) and the third order 3A_1_(LO) can observed as well, at 1700 cm^−1^. There is an increasing background intensity caused by the PL (NBE emission band) of ZnO. The Raman bands show a clearly asymmetrical shape. The 400–700 spectral region after PL background correction by a linear baseline is shown in Figure 8b,d, along with the deconvolution of the main band corresponding to the A_1_(LO) mode, revealing a main contribution centered at 570 cm^−1^, and an additional wide band that can be assigned to different optical–acoustic combination modes, such as the E_2_^high^ mode, associated with the vibration of oxygen atoms, which is usually the main Raman band observed in the case of visible excitation Raman, also the E_1_ transverse optical (TO) and longitudinal optical (LO) phonon modes. The sharp spike at ~521 cm^−1^ arises from the contribution of the Si substrate. Raman peaks for AZO film become broader compared with pure ZnO, due to disorder, strain and defects introduced in the crystal quality by doping. The position and width of the A1 Raman mode of AZO films deposited on Si is not significantly affected, compared to the resonant Raman spectra of ZnO films deposited on Si or to the AZO films deposited on glass [52].

#### 3.3.3. PL Analysis

**Figure 9:**
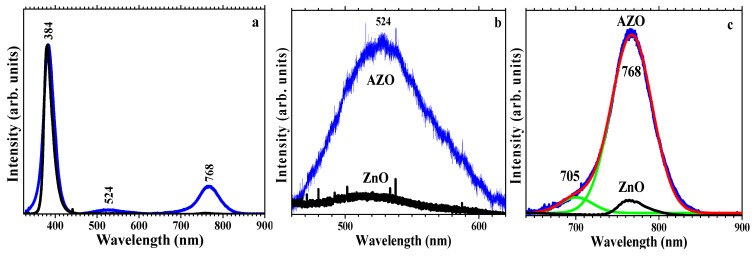
PL spectra of ZnO (black) and AZO (blue) thin films deposited on Si (**a**), detail of the green emission band (**b**) and deconvolution (green curves) of the red emission band for the AZO film (**c**).

The PL emission spectra (λex = 325 nm) were performed at room temperature and show a strong UV emission at ~384 nm that corresponds to the near-band-edge (NBE) emission of ZnO due to the radiative recombination of free excitons [72], a relatively weak deep-level (DLE) green emission band at ~524 nm, originated from the transition from the conduction band to deep defect levels, assigned to zinc vacancies occupied by oxygen antisites, O_Zn_, in the AZO thin film [73] and a red band ~768 nm that corresponds to the second order of the NBE band [74] (Figure 9). The strong second order NBE band hides the smaller band corresponding to deep-level red emission at 705 nm. The undoped films do not present the green (524 nm) and red (705 nm) emission bands. When compared with the PL emission bands from AZO films deposited on glass, we observe that the NBE band is both better localized and red-shifted to higher wavelengths (from 376 nm to 384 nm) [75]. Besides the sharpening and higher intensity of the NBE band, a strong second order is also observed in the film on Si substrate and not in AZO films deposited on glass. The sharpening of the NBE can indicate a more ordered lattice in the films deposited on the Si substrate.

Besides the sharpening and higher intensity of the NBE band, a strong second order is also observed in the film on Si substrate and not in the AZO films deposited on glass, as well as an additional contribution in the blue that, according to Lin et al. [76], should be attributed to zinc vacancies V_Zn_. The sharpening of the NBE and the absence of blue emission indicate a more ordered lattice in the films deposited on the Si substrate.

A comparison between the emissions bands of our AZO films and those from the literature is presented in Table 4.

As observed, the PL properties vary by the modification of several parameters as the reaction solvent type, the substrate used, the thickness of the films and the number of layers or dopant concentration. Moreover, the spin or dip-coating deposition can affect the PL properties as reported in the literature [77,78]. In their related papers [29,52] regarding the PL of AZO thin films prepared using the sol–gel technique, Kumar et al. demonstrated that by using the same solvents (2-methoxyethanol, methanol, ethanol and isopropanol) and substrate (glass) but changing the deposition method (spin or dip-coating), some modification of the peak intensities occurred. By comparing the PL spectra of spin or dip-coated samples, they display similar emission peaks in the UV and visible regions, but with different intensities. As a consequence, for the case of spin-coated samples, the best results in terms of PL properties were noticed for isopropanol, while for the dip-coated samples, the methanol was more efficient.

The effect of Al^3+^ incorporation in the ZnO thin films onto the PL properties was also investigated by Sandeep [53] and Srinatha [54]. The spin-coated AZO thin films reported in [53] prove to exhibit PL behavior in a steady state with the blue emission due to the defects-related transitions as a result of Al doping, which also indicates a decrease in the film quality. The PL studies described in [54] showed that the amount of dopant (Al) had an Influence on the PL intensity (see Table 4). The PL spectra display UV (362 nm) and near UV emission (408 nm) bands assigned to the NBE emission and a visible emission attributed to defect-related deep-level emissions. It was concluded that the density of defects in the AZO film is reduced in comparison with the pure film, which is related to a different dopant concentration.

To the best of our knowledge, there are few recent literature papers concerning the PL of sol–gel AZO thin films deposited on Si substrate [55]. In contrast, our results are similar to those reported by Lee and colleagues [55], with the emission bands being shifted to lower or higher wavelengths, except for the band from 768 nm corresponding to the second order of the NBE band, as already mentioned in the chapter. These differences could be assigned to another deposition method, such as the dip-coated (present work) and spin-coated [55] methods, and also to the different numbers of layers, 10 (our work) and 7 [55], respectively.

Additionally, in our case the green emission band can be correlated with a higher crystallinity of the AZO thin film and an increased O/Zn ratio. Our XRF compositional results detect excess oxygen, results that exclude a priori the presence of oxygen vacancies and highlight the presence of interstitial oxygen and/or oxygen antisites. The observed green emission must correspond to the defects associated with the O/Zn ratio greater than one, including Zn vacancies, V_Zn_, oxygen interstitials, O_i_, or oxygen antisites, O_Zn_. The energy gap between the conduction band and the O_Zn_ level (2.38 eV) is exactly consistent with the position of the green emission band (521 nm) [76]. The energy of the O_i_ level (~2.28 eV) is also close to the green, toward orange-red emission, but the probability of forming O_i_ is small due to the large diameter of the oxygen atom, and the energy gap, 3.06 eV of V_Zn_, is too large to be related to the green emission. Oxygen vacancies are more likely associated with red emission at longer wavelengths due to their lower energy levels (~1.6 eV), while the origin of the purely green PL emission band of AZO at 520 nm must be assigned to deep-level O_Zn_ lattice defects.

## 4. Conclusions

ZnO and AZO thin films were prepared using the sol–gel method and deposited on Si(100) substrate using the dipping technique. The structure, morphology, optical properties and deep-level defects were assessed using a combination of complementary techniques.

XRD proved that the films are polycrystalline with a hexagonal wurtzite structure, while the XRF method highlighted the presence of Al in the ZnO matrix for the AZO films. The slightly lower lattice parameters in comparison with the standard ZnO suggest the replacement of Zn^2+^ by Al^3+^ during the formation of the AZO film. The AZO film thickness was around 300 nm as estimated from the SEM and SE. From a morphological point of view, the AZO film shows microscale irregular aggregates, typically 1–2 microns which consist of nanoparticles with sizes around 50 nm, in agreement with the AFM and SEM analyses. Meanwhile, the ZnO film consists of nanoscale grains alternating with nanosized intergranular porosity. AFM showed that the roughness of the AZO film is ~3 times higher than the undoped ZnO. The SE parameters (thickness, refractive indices and optical band gap) increase after doping, e.g., the refractive index increased from 1.78 to 1.89 (at 630 nm) and the band gap increased from 3.42 to 3.45 eV, which is most likely related to a better densification and an enhancement in charge carrier density, respectively.

The PL spectra showed a strong UV emission corresponding to the near-band-edge emission of ZnO. The effect of the defects introduced by the Al dopant into the ZnO lattice in the AZO thin film was evidenced by a pure green deep-level emission band at 520 nm attributed to O_Zn_ lattice defects.

## Figures and Tables

**Table 1:** Elemental composition of AZO film deposited on Si substrate.

Element	Mass %	at. %	Det. Limit	El Line
O	22.91	54.63	1.69951	O-KA
Al	0.46	0.65	0.11966	Al-KA
Zn	76.63	44.72	1.57686	Zn-KA

**Table 2:** Parameters obtained from XRD analysis.

Sample	2θ (100) (°)	FWHM (°)	2θ (002) (°)	FWHM (°)	2θ (101) (°)	FWHM (°)	Lattice Parameters (Å)		Average Crystallite Size (Å)
							a = b	c	
ZnO film	31.80	0.730	34.45	0.711	36.33	0.842	3.253	5.211	115
AZO film	31.77	0.488	34.58	0.329	36.43	0.443	3.241	5.194	212
ZnO 36-1451	31.77	-	34.42	-	36.25	-	3.250	5.207	-

**Table 3:** Parameters of AZO/Si vs. ZnO/Si films determined using SE analysis.

	D_film_ (nm)	d_rough_ (nm)	n (λ = 630 nm)	MSE	E_g_ (eV)
AZO	332.1	12.4	1.89	8.2	3.45
ZnO	174.6	5.4	1.78	10.4	3.42

**Table 4:** Room temperature PL properties of AZO thin films deposited using sol–gel method.

Substrate	Dopant Conc.	Emission Features Depending on Various Parameters	Ref.
Glass	3 mol %	UV emission (NBE): 387 nm; visible emission: 415 (blue) and 483 nm (blue green); effect of solvent on PL intensity: the best results were obtained for isopropanol.	[52]
Glass	2 at. %	UV emission (NBE): 386 nm; visible emission: 413, 436 (blue) and 481 nm (blue green); effect of solvent on PL intensity: the best results were obtained for methanol.	[29]
Glass	3 wt %	UV emission (NBE): 383 nm; visible emission: 420 nm (violet), 435, 467 nm (blue); Al doping: increase in luminous efficiency (AZO: 22.8%; ZnO: 10.8%) as a result of blue emission enhancement.	[53]
Glass	0–3 mol %	UV emission (NBE): 362; near UV emission: 408 nm (NBE); visible emission: 450, 483 nm (blue emission); 530 nm (green emission); 1–2 mol% Al doping: decrease in NBE peak intensity; 3 mol% Al doping: increase in PL intensity.	[54]
Glass	3 at. %	UV emission band: 396 nm (blue); visible emission: 418 (violet), 440, 450, 467 nm (blue), 480, 493 (blue green), 547 nm (green), 589 nm (yellow); PL intensity decreases with the increase in thickness of the film as follows: 6 > 9 > 12 layers.	[31]
Glass	0–5 wt %	NBE: 370 nm; visible emission: 413 nm (violet), 487 nm (blue), 531 nm (green), 630 nm (orange-red); PL intensity increases as follows: 5 < 1 < 3 wt% Al. This orange-red emission appears because of Al doping which leads to a large number of defects in ZnO film.	[56]
Si	0–3 vol %	UV emission: 365 nm; visible emission: 530 nm; the PL intensity of visible peak decreases with the increase in Al concentration (vol%) as follows: 3 < 2 < 1 (vol%). The corresponding visible emission band was red-shifted from 500 nm to 530 nm upon Al introduction.	[55]
Si	0.46 at. %	UV emission (NBE): 384 nm; visible emission: 524 nm (green); 768 nm (red)	This work

Note: NBE—near-band-edge emission; λex (nm): 325 for [29,31,52,53,54,55]; 320 [56].

## Data Availability

The data reported in this research are available from the corresponding authors upon request.
